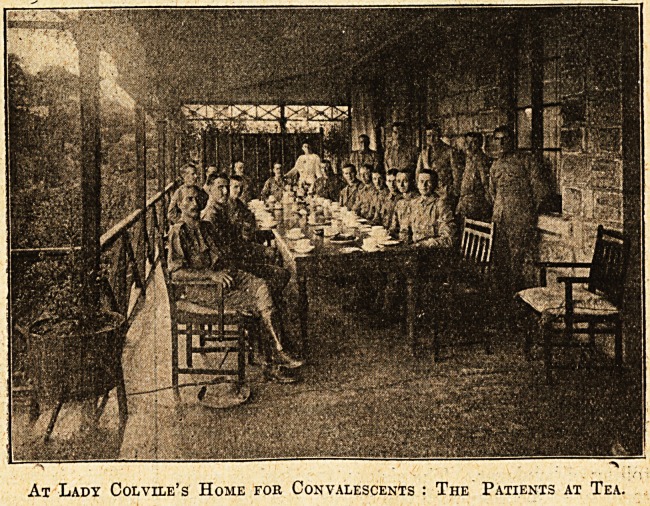# Hospital Work in East Africa: Kikuyu and Limoru

**Published:** 1916-12-16

**Authors:** 


### HOSPITAL WORK IN EAST AFRICA.

### Kikuyu and Limoru.

Hospital work in East Africa is carried on with difficulty. Although only a Colony it embraces every form of climate, while the distances are enormous. Indeed, taking in Uganda, it is well over one thousand miles as the crow flies from Mombasa to Kabarega, while the actual frontier along which the forces face each other at the joint boundary is six hundred miles in length. Consequently there have to be a number of base hospitals as well as convalescent establishments.

Of the base hospitals there are already seven, and there may be more as the operations extend.

One of the Bedrooms for Officers at the Maharajah Scindia of Gwalior Convalescent Home at Kikuyu.

The two chief convalescent homes are the one founded by Lady Colvile in her house near Limoru and the other at Kikuyu, near Nairobi, financed and fitted out entirely at his own expense by the Maharajah Scindia of Gwalior.

The Limoru institution is for Europeans, and the Gwalior Home at Kikuyu for Indians, of whom there are a large number in the Colony, mostly Imperial Service troops from Kashmir and other leading Native States. The Lady Colvile Home accommodates about twenty-four soldiers, while the Gwalior can house almost twice the number, as it is rather larger.

Both institutions are situated on high ground, have delightful surroundings, and are well managed. They are elaborately fitted out, since the Maharajah has spent a large sum of money, while Lady Colvile, whose son, Mr. Gilbert Colvile, is a settler in East Africa, has enjoyed the assistance of friends, though the bulk of the administrative work has fallen on her shoulders.

The Gwalior Home consists of a religious house built by French missionaries, fitted out for Indian patients, and a small Home some distance away which accommodates about six English officers. The officers' Home was built for a sanatorium and taken over when the war broke out for military use; it is therefore admirably suited for the purpose. There are fully qualified English nurses and a masseuse on the staff.

Maharajah Scindia of Gwalior Convalescent Home at Kikuyu. A Group of the Staff.

The medical staff is European as regards the highest control, but all the routine work is carried out by natives. The cases are mostly due to actual wounds, but there is a certain amount of tropical disease. As a rule recovery is rapid, owing to the purity of the air at the considerable elevation.

At Lady Colvile's Home for Convalescents: The Patients at Tea.

## Figures and Tables

**Figure f1:**
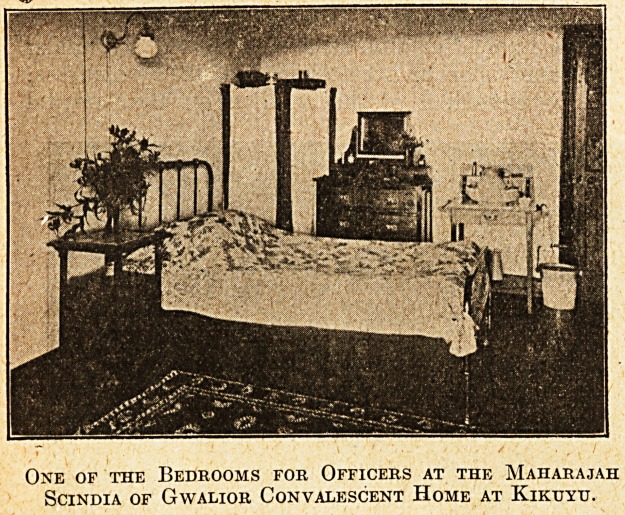


**Figure f2:**
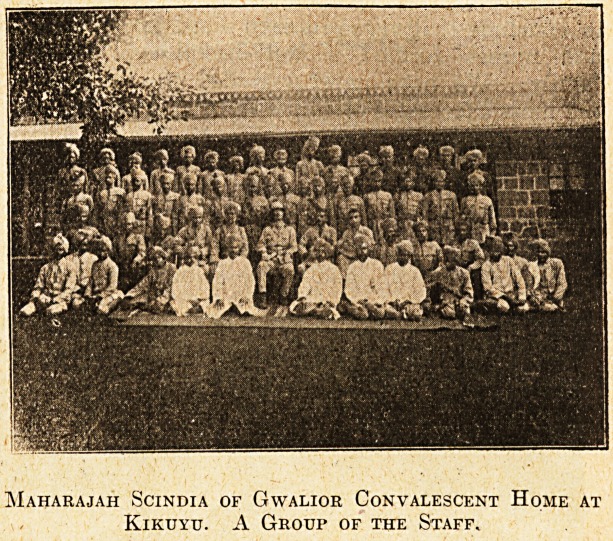


**Figure f3:**